# Eviscération utero cutanée des suites opératoires d’un myome utérin

**DOI:** 10.11604/pamj.2016.25.137.10362

**Published:** 2016-11-04

**Authors:** Traore Abdoulaye Ababacar, Alaoui Lamrani Youssef

**Affiliations:** 1 Service de Radiologie du CHU Hassan II Fès, Maroc

**Keywords:** Eviscération, utérus, fistule, Operated uterine myoma, utero-cutaneous evisceration, CT scan

## Image en médecine

Il s’agit d’une patiente de 42 ans, deuxième geste, deuxième parité, admise pour prise en charge d’un myome utérin interstitiel. Les suites opératoires ont été marquées par une infection cutanée associée à une éventration utérine. A l’examen, la patiente était fébrile à 39.5°C, associée à une éviscération de l’utérus avec issu de pus verdâtre à travers le site opératoire. Le bilan biologique réalisé montrait un taux d’hémoglobine à 12g/dl, une hyperleucocytose à polynucléaire neutrophile 15000/uL et la C Réactive Protéine à 320 mg/l. Ces résultats ont conduit à la réalisation d’un complément TDM qui a montré une fistule utero-pariétale avec une éviscération utérine, sans complication digestive associée. L’utérus était également disroté avec un contenu hydro-aérique, faiblement rehaussé en périphérie après contraste, confirmant une surinfection associée. La prise en charge chirurgicale a été effectuée avec curetage de la cavité utérine à travers la brèche de perforation qui a été par la suite suturée. Ce cas met en exergue une complication rare de perforation utérine dans les suites opératoires d’une myomectomisse utérine.

**Figure 1:**
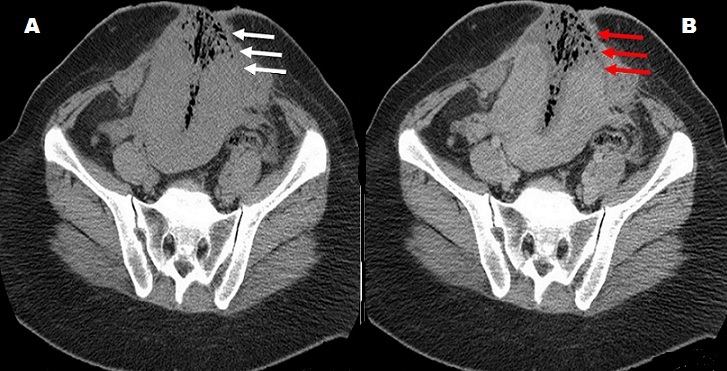
Coupes TDM axiales en contraste spontané (A) et après contraste (B) montrant une fistule utero-pariétale avec une éviscération utérine (flèche blanche). L’utérus avait un contenu hydro aérique faiblement rehaussé en périphérie après contraste, en faveur d’une surinfection (flèche rouge)

